# Microcircuits neuroscience to understand pathophysiology

**DOI:** 10.18632/oncotarget.14792

**Published:** 2017-01-21

**Authors:** José Bargas, Jesús Pérez-Ortega

**Affiliations:** División de Neurociencias, Instituto de Fisiología Celular, Universidad Nacional Autónoma de México, México City, México; División de Neurociencias, Instituto de Fisiología Celular, Universidad Nacional Autónoma de México, México City, México

**Keywords:** functional histopathology, living tissue, bioassays, network analysis, striatum, Neuroscience

The histological scale is the preferred level for a large number of anatomical, pathological, pharmacological, immunochemical, and molecular studies. However, neurophysiological studies are mostly performed at the cellular/molecular or systems levels. The former describes the biology of the nerve cells and their synapses considering the genetic and signaling paths that underlie neural function while the latter involves the localization of cerebral processes correlating neural activity with sensation, motor coding, emotions and cognition. All levels are necessary to build a complete picture of any organ more so in the case of the brain. But there is no easy way to connect the cellular/molecular and systems levels without intermediate stances [1]. Even though, with the use of high technological tools, more recent work claims to do so: from molecules to behavior. The result has been a large amount of data: several different molecules or cellular processes are being proposed as potential causes of the same behavior, disease or circuit malfunction [1]. One example is Parkinson’s disease (PD), where it is known that animal models with dopamine deprivation end up with several synaptic and neuronal dysfunctions in different brain nuclei and circuits, and although all of them claim to be correlated with the disease, none of them can be said to be the single most important disorder that causes it, except for dopamine deprivation itself. It is hard to connect all these disorders in a single unifying theory that explains PD signs and symptoms. In the same way it is common place to correlate synaptic plasticity of a particular connection with learning and memory of a whole animal.

Since Sherrington, Brown, Hebb, Mouncastle and others, it has been posited that mammalian neurons do not work alone but do so in modular arrangements or microcircuits that iterate in the brain with certain variance. In trying to begin fill the gap between cellular and systems levels we thought that the histological scale deserves to be explored physiologically to try to understand how a neuronal microcircuit conformed by several neurons and synapses works. Methods to visualize and record the activity of dozens of cells simultaneously are available and are improving. Here, we used calcium imaging. What has been lacking is a way to describe and quantify the activity of a microcircuit in the same way that, at the cellular level, neurophysiologists characterize single neurons or synapses with measures such as input resistance, spike rate, time constant, quantal content, short-term dynamics, etc.

In Pérez-Ortega et al. (2016) [2] we propose that measures taken from Graph Theory also called Network Theory can be used to describe and quantify the activity of a neural microcircuit, then a graphical visualization and quantitative description was devised by choosing a small set of parameters [2]: under control conditions neuronal ensembles consisted in clusters of neighboring cells connected with other clusters at distances that could not be explained by monosynaptic connections but needed the action of long axon neurons or interneurons. These “hub” neurons were highly connected and showed the principle of reciprocal innervation between neuronal ensembles. As hypothesized by Sherrington and Brown, reciprocal innervation may explain alternation of activity between ensembles which could be observed as the ordered, hierarchical and cyclic activation of neuronal ensembles. Thereafter we asked whether this technique can be used to describe the circuit under pathological conditions such as Parkinsonism or L-DOPA induced dyskinesias (LID). The analyses readily showed differences between control and pathological conditions and among pathological conditions themselves. What surprised us most was the almost metaphorical description that arose. As some investigators had inferred before [3] and fMRI analyses have shown for the whole brain [4]: corticostriatal connections were severely altered in Parkinsonian microcircuits while “hub” neurons had significant changes in function. Moreover, during LID, when movement is known to come back (“on” state), “hub” neurons returned in significantly larger numbers than in controls, albeit the trajectories of ensembles activity became disordered loosing hierarchical and cyclic behavior. Now we are using transgenic animals and optogenetics to identify the different actors of these microcircuits, and at the same time we are exploring other brain regions.

**Figure 1:**
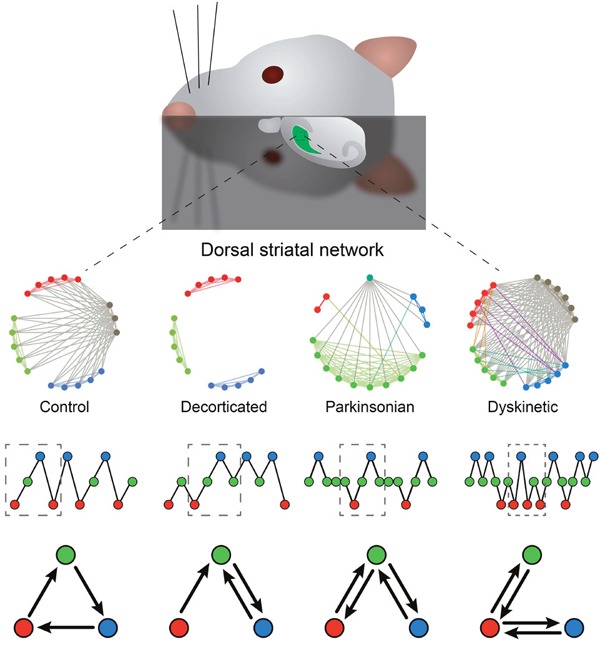
Overview of striatal microcircuit analyses Top: Rodent brain slices including the dorsal striatum were used. Top row illustrates differences in the topological representations of control, decorticated, parkinsonian and dyskinetic microcircuits. Control tissue exhibits a network with reciprocal innervation between neuronal ensembles (colored) by means of “hub” neurons (grey) which is lost in decorticated preparations and nearly lost in parkinsonian tissue. “Hub” neurons reappeared in larger numbers in the dyskinetic circuit. The second row shows the time course of ensembles activity trajectories and the third row shows a spatial representation of these trajectories. Note balanced alternation between ensembles and cyclical trajectories in control circuits. In contrast, decortication produces unbalanced and incomplete trajectories. This finding was also true for the parkinsonian circuit where one ensemble gets most neurons (green) and only one or a few hub neurons remain. During LID reappearance of hub neurons did not result in balanced and complete circuits.

We would like to emphasize that neither calcium signaling nor the modular architecture are a property of excitable cells only. Liver lobules, pancreatic islets, alveoli, kidney glomeruli and tubules, motor units, etc. show that cells work together to form higher level composites. Accordingly, calcium imaging may perhaps be used to analyze living tissue from biopsies and then see the functional alterations that different pathologies represent. This means that both histology and pathology may begin to be re-written in a functional living way and new methods of diagnosis and prognosis could be available in the near future. Novel pharmacological bio-assays may also be devised [5].
